# Langerhans Cell Histiocytosis Masked by Constipation: A Case Report and Literature Review

**DOI:** 10.7759/cureus.69671

**Published:** 2024-09-18

**Authors:** Alexis N Reinders, Farrah Gaston, Haroon Ali, Michele K Beekman, Prerna Kumar

**Affiliations:** 1 Pediatrics, University of Illinois College of Medicine, Peoria, USA; 2 Internal Medicine - Pediatrics, University of Illinois College of Medicine, Peoria, USA; 3 Pediatrics, University of Wisconsin-Madison, Madison, USA; 4 Pediatric Hematology and Oncology, University of Illinois College of Medicine, Peoria, USA

**Keywords:** bone lesion, gastrointestinal (gi), langerhans cell histiocytosis (lch), pediatric constipation, pediatric oncology

## Abstract

Langerhans cell histiocytosis (LCH) is a rare histiocytic neoplastic disorder that presents in all age groups, although it often affects young children. Patients typically present with lytic bone lesions and an erythematous rash, though other systems such as the digestive, endocrine, lymphatic, and respiratory systems can be involved.

We present a case of LCH that was masked by symptoms of constipation. The patient was a three-year-old female who presented with a primary complaint of constipation accompanied by abdominal and back pain. Further investigation identified an L3 lesion on lumbar spine magnetic resonance imaging, for which a pediatric neurosurgeon performed an open reduction and internal fixation. Pathology confirmed the diagnosis of LCH. This was followed by one year of chemotherapy. To date, she has not had a recurrence of LCH.

This case demonstrates the importance of generating a broad differential diagnosis and determining and treating the etiology of a patient’s symptoms rather than the symptoms alone. Physicians must maintain a high index of suspicion for rare diagnoses when symptoms have persisted and more common etiologies have been ruled out. A thorough neurological exam should be performed for all patients with constipation due to an unknown etiology, especially when accompanied by back pain. Although the patient did not present with gastrointestinal (GI) involvement of LCH, nonspecific GI symptoms such as diffuse abdominal pain and bloody diarrhea have been associated with this rare diagnosis. We thoroughly review the literature regarding both GI involvement of LCH and cases of LCH that present with accompanying GI symptoms. Additionally, we highlight the clinical treatment options of LCH.

## Introduction

Langerhans cell histiocytosis (LCH) is a rare condition characterized by the abnormal proliferation of neoplastic cells, which are derived from myeloid dendritic cells and resemble dendritic antigen-presenting cells, otherwise referred to as Langerhans cells [1,2]. Approximately half of the patients with LCH have a BRAF-V600E mutation, which constitutively activates the MAPK/ERK pathway. Other mutations have been identified including MAPK21, ARAF, and ERBB3; the knowledge that these mutations drive disease supports why LCH is now considered a myeloproliferative neoplasm derived from hematopoietic stem cells and myeloid precursors [3-5].

From an epidemiological standpoint, LCH may present in any age group, although it more commonly presents in childhood [6]. The incidence in children has been approximated to be as high as five cases per one million and decreases with age [2]. In children less than 15 years of age, the incidence is four to five cases per million per year, while the incidence is one to two cases per million per year for those greater than 15 years of age and even lower in adults [2,6]. LCH has been shown to be more common in males [2,7]. 

LCH most commonly occurs in the bones, and often clinically presents as a pathological fracture, localized pain and swelling, or as an incidental finding on imaging [7]. Rarely, LCH of the bones presents as constipation due to spinal cord compression, resulting in impaired bowel function [8]. 

True gastrointestinal (GI) involvement is extremely rare in LCH, can present at any age, and may present with vomiting, failure to thrive, protein-losing enteropathy, hypoproteinemia, anemia, diarrhea, constipation, hematochezia, and perianal lesions [9,10]. Those presenting with GI involvement of LCH (GI LCH) are often young children under the age of 18 months. In one review of 24 patients with LCH involvement of the GI system, the majority of the patients had multi-system involvement at the time of diagnosis including skin (86%), liver (80%), bone (23%), and lungs (14%); the most common presenting GI symptoms in this cohort were hypoproteinemia (77%), hematochezia (59%), and non-bloody diarrhea (18%), followed by rarer symptoms of constipation (9%) and perianal fistulas (4%) [11]. In another review, approximately 2% of the patients with GI LCH presented with malabsorption or diarrhea [2].

In this report, we review the existing literature regarding both GI involvement of LCH and cases of LCH that present with accompanying GI symptoms.

## Case presentation

A previously healthy, developmentally appropriate, three-year-old female with no prior history of constipation initially presented to the emergency department (ED) with a three-week history of constipation accompanied by occasional severe abdominal and lower back pain. Upon further questioning, she ate a normal, varied diet but did show hesitation with stooling at daycare. The patient had been receiving MiraLAX and Pedia-Lax with minimal stool output, and the last bowel movement was noted to have been the morning prior to her presentation to the ED. To assess the extent of constipation, an abdominal X-ray (XR) was ordered, which showed a non-obstructive bowel gas pattern with an average-to-large amount of stool in the colon. Additionally, her parents mentioned she recently had a fall. To assess the back pain in the setting of a recent fall from a height of two to three feet, a lumbar spine (L-spine) XR was performed, which did not demonstrate significant findings. The patient’s parents were advised to increase the dose of MiraLAX and follow up with their pediatrician. 

Four days later, the patient returned to the ED with similar complaints as before but additionally was refusing to eat. The patient was afebrile with normal vital signs. Pertinent negatives included fever, nausea, vomiting, hematuria, and dysuria. The patient received a saline enema; however, only a small amount of stool was evacuated as a result. 

The patient was transferred to our hospital to be admitted for a trial of GoLYTELY. On arrival, the patient was afebrile with stable vital signs. A nasogastric tube was placed and a single-view abdominal XR, abdominal ultrasound (US), lumbar XR, and hip XR were performed. There were no significant abnormalities noted in any imaging. A complete blood count, comprehensive metabolic panel, C-reactive protein, thyroid stimulating hormone, and lipase were all performed and were within normal limits. The erythrocyte sedimentation rate was mildly elevated. 

**Figure 1:**
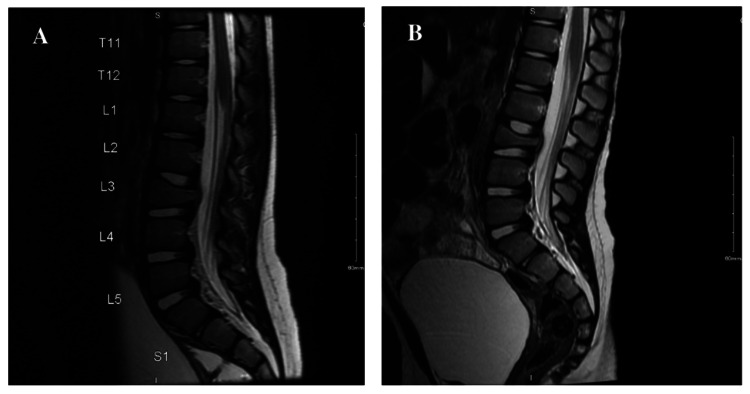
Initial and follow-up L-spine MRI A) Sagittal T2 sequence from the initial L-spine MRI demonstrating altered T1 marrow signal at the L3 vertebral body and posterior elements, significant height loss of the posterior aspect of the L3 vertebral body, and extraosseous involvement at the anterior epidural region with at least minimal/mild thecal sac encroachment. B) Sagittal T2 sequence from follow-up L-spine MRI eight days later demonstrating mild progression of the L3 vertebral body compression deformity.

The patient’s back pain progressed to the point where she was refusing to bear weight. The pain was now reported to be band-like at the L3 level, and on physical exam, the patient was noted to have decreased muscle strength in her bilateral lower extremities, with 3+ in her right lower extremity and 4+ in her left lower extremity. No other focal neurological findings were identified. Muscle tone and bulk were normal for age. Due to these new and concerning symptoms, magnetic resonance imaging (MRI) of the brain and cervical, thoracic, and lumbar spine were performed. MRI was significant for an altered T1 marrow signal at the L3 vertebral body and posterior elements, significant height loss of the posterior aspect of the L3 vertebral body, and extraosseous involvement at the anterior epidural region with at least minimal/mild thecal sac encroachment (Figure 1A). The differential diagnosis included an indolent, infectious process versus a neoplastic process, for which Pediatric Neurosurgery, Neurology, Infectious Disease, and Hematology/Oncology were consulted. 

A thorough workup was immediately initiated to identify the underlying etiology of the L3 lesion. Neither the history nor the imaging findings were strongly suggestive of a traumatic cause for this lesion, despite the recent fall from a height of two to three feet. The focal and isolated nature of the MRI findings suggested a more insidious process. Thus, an extensive workup for a concerning infectious etiology was conducted including tuberculosis, Bartonella, Brucella, Histoplasma, Aspergillus, and aerobic, anaerobic, and fungal blood cultures. Peripheral blood smear, urine homovanillic acid, and urine vanillylmandelic acid were obtained to evaluate for oncologic etiologies including leukemia and neuroblastoma. The workup was unremarkable. 

Three days later, Interventional Radiology performed a fluoroscopically-guided left L3 vertebral body biopsy; unfortunately, the results were non-diagnostic. Five days later, a follow-up L-spine MRI was performed which showed mild progression of the L3 vertebral compression deformity with similar ventrolateral epidural soft tissue spread and mild spinal canal stenosis (Figure 1).

**Figure 2:**
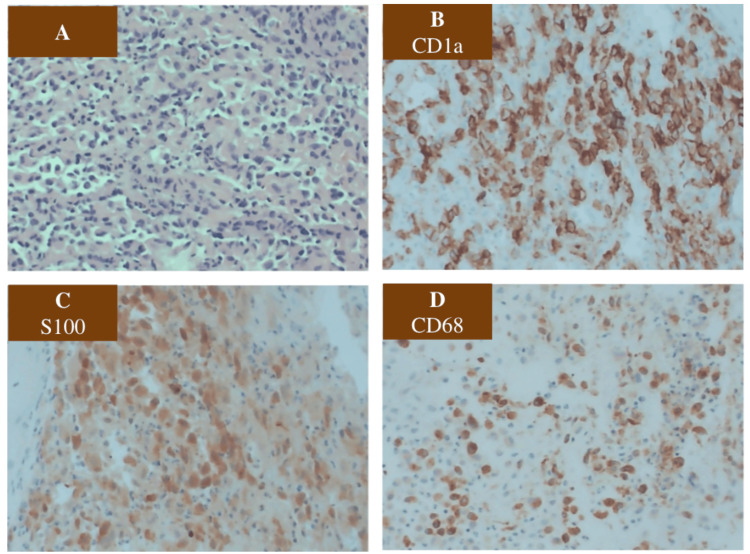
Histopathology of Langerhans cell histiocytosis (LCH) A) Portion of the L3 vertebral body removed during laminectomy demonstrated sheets of histiocytes with elongated, irregular nuclei infiltrating the marrow space at 100x magnification. B, C, D) Immunohistochemistry positive for CD1a, S100, and CD68, which confirmed the diagnosis of LCH.

The following day, Pediatric Neurosurgery took the patient to the operating room for open reduction and internal fixation of the lesion. Surgical pathology results confirmed the diagnosis of LCH (Figure 2). Three days later, the patient was discharged home in stable condition. Pending pathology results, the patient was scheduled for outpatient appointments in the Pediatric Hematology/Oncology and Neurosurgery clinics. Pediatric Infectious Disease opted to follow the patient peripherally due to the likely neoplastic etiology. 

The patient’s case was discussed at our institution's multidisciplinary pediatric tumor board. Staging confirmed single-system unifocal LCH with involvement of the L3 vertebral body only. 

No further surgical intervention was needed. Systemic chemotherapy was initiated as a post-operative maintenance regimen and consisted of oral methotrexate and 6-mercaptopurine (6-MP). Trimethoprim-sulfamethoxazole was started for *Pneumocystis jirovecii* prophylaxis. Chemotherapy was well tolerated with only mild back pain and decreased appetite. The chemotherapy regimen was continued for one year with no other complications, at which time it was discontinued. Trimethoprim-sulfamethoxazole was empirically continued until the patient had been off therapy for three months. Off-therapy surveillance laboratory tests and imaging, including skeletal survey and abdominal US that showed no evidence of recurrence, were done every three months for the first year and gradually spaced out after that. The patient was seen by physical therapy intermittently during treatment and was discharged with no restrictions on mobility or other concerns. To date, the patient remains in remission, approximately three and a half years after the completion of therapy. 

## Discussion

Our case highlights a presentation of LCH in a three-year-old patient with back pain masked by constipation. In the absence of GI involvement and spinal cord compression, there have been no other case reports of LCH presenting with constipation. 

In this case, the patient's back pain was confirmed to be due to a vertebral lesion. The differential diagnosis is broad and can include many diagnoses including musculoskeletal, infectious, neoplastic, and autoimmune etiologies. Musculoskeletal etiologies that can present with vertebral body abnormalities include trauma with vertebral body compression or fracture, cystic bone lesions, and intervertebral disc calcification. Infectious etiologies driving abnormal vertebral body and spine findings on imaging include discitis, osteomyelitis, abscesses, tuberculosis including Pott's disease, fungal disease, and transverse myelitis. Oncologic etiologies that can present with vertebral body and spine abnormalities include Ewing sarcoma, neuroblastoma, osteosarcoma, leukemia, lymphoma, and LCH. Our patient was ultimately found to have a spinal lesion on MRI, which was later biopsied and found to be LCH.

System involvement, prognosis, and common clinical management

LCH may present with single-system or multi-system involvement (MSI), with the latter often carrying a poorer prognosis. Single-system involvement (SSI) may include one or more lesions affecting the same organ systems (uni-focal versus multi-focal disease), while MSI includes more than one organ system. The involvement of the central nervous system (CNS), lungs, liver, spleen, and bone marrow are all associated with a poor prognosis [1,2].

Approximately half of the children with LCH have MSI, which is most common in those less than three years of age, while SSI is more common in older children and adults [2]. Clinical symptoms at presentation depend on the organs involved and may include bone pain, lymphadenopathy, hepatosplenomegaly, and new-onset diabetes insipidus. The most common organ systems involved in LCH are bone, skin, lymph nodes, liver, spleen, lung, and the CNS [1,2]. Our patient was ultimately found to have SSI with bone involvement only, leading to an overall good prognosis.

When LCH affects bone, pathological fractures, localized pain and swelling, and incidental lesions on radiography are common presenting findings [7,11]. In one 2009 study, multifocal bony lesions were compared based on SSI (n=67) versus MSI (n=97) in children with an average age of two years. The most common bony lesions were found to be in the skull and spine in both groups. The most common skull bones affected were the temporal bone followed by the petrous, parietal, frontal, and occipital bones. The spine was the second most commonly affected group of bones with both groups reporting the most lesions in the lumbar vertebrae followed by the thoracic, cervical, and sacral vertebrae. The third most common group of bones affected were the lower extremity long bones (femur > tibia > fibula) in both groups [12]. Our patient had LCH of the lumbar spine with involvement of the most commonly affected region [1,2].

As for clinical management, treatment for LCH varies and can consist of curettage with intralesional steroids for a single lytic bone lesion, oral chemotherapy with 6-MP and methotrexate for single-system skin LCH, or a combination of intravenous vinblastine and oral prednisone for some patients, with the additional need for cytarabine, clofarabine, or rituximab in others [5]. Targeted therapies such as trametinib and dabrafenib are directed at the MAPK/ERK pathway and are a recent advancement in the treatment of LCH [13]. Ongoing surveillance is required for patients with LCH after completion of therapy due to the risk of recurrence, complications from the disease, and late effects of therapy. Complications vary based on the extent and location of the disease and can include growth and developmental delay, musculoskeletal deformities, decreased lung function, ascending cholangitis potentially requiring transplant, and dental problems [5]. Our patient was ultimately treated with oral methotrexate and 6-MP for unifocal single-system LCH. Intralesional steroids were not given at the time of the biopsy due to an unclear diagnosis and lack of suspicion for LCH. The patient remains in remission after completing one year of treatment.

Uncommon clinical management: radiation therapy (RT)

Although rare, RT has been utilized in the treatment of LCH, either alone or as an adjuvant therapy to surgery and/or chemotherapy. Reported LCH cases treated with RT have used 10.5-20 Gy in 6-10 fractions with techniques ranging from electron beam RT to intensity-modulated RT. In a review of 39 patients with a total of 46 LCH lesions, 15 of which were from pediatric patients, RT was shown to more readily prevent local recurrence in isolated bone LCH when compared to non-bone LCH. In fact, at a three-year follow-up, 63.2% of patients with non-bone LCH had no local recurrence, while there were no reported local recurrences in patients with bone LCH. The rate of recurrence may also be related to the underlying location of such tumors rather than the treatment itself. RT has also been shown to play a role in refractory LCH. Nine of the 46 lesions treated with RT had previously been refractory to other therapeutic regimens. Though this study did include pediatric patients, it was a small retrospective study. More research is needed to evaluate both the efficacy and long-term risk profile in children prior to routine use in clinical practice [14]. Accordingly, RT was not a considered therapy for this pediatric patient with LCH. 

LCH with GI involvement

When discussing a case of LCH masked by constipation, it is important to distinguish this from GI LCH, which can also present with constipation. Nonspecific GI symptoms such as hematochezia, constipation, diarrhea, and abdominal pain have commonly been reported in the setting of gastrointestinal LCH, which represents less than 3% of the cases [9]. Although rare, patients with LCH who present with gastrointestinal involvement typically have MSI at the time of diagnosis, with involvement of other more common sites of disease such as skin, lymph nodes, and liver. Though single-system disease can be associated with good outcomes and complete remission, multi-system LCH is more often associated with a chronic relapsing-remitting disease. Various reports suggest that multi-system LCH carries a 20% mortality rate [6,7,11,15]. Cases of GI LCH and LCH masked by constipation are both rare.

One study described a 22-month-old female who presented with frequent vomiting, occasionally bloody diarrhea, fever, and generalized swelling. A physical exam revealed a distended abdomen with erythematous lesions and hepatosplenomegaly, while a laboratory workup demonstrated anemia, hypoalbuminemia, and hypertriglyceridemia. An abdominal computed tomography (CT) scan was performed, which showed an edematous small bowel, which prompted an upper GI endoscopy followed by a colonoscopy. The upper GI endoscopy revealed an ulcer at the second part of the duodenum, while the colonoscopy showed ulcers and erythematous lesions throughout the sigmoid colon, which were consistent with LCH on histologic review. The patient was treated with antibiotics, albumin infusions, total parenteral nutrition, and intravenous methylprednisolone followed by oral prednisolone, vinblastine, and 6-MP. The patient was reported to have no signs of active disease one year following completion of therapy [9].

Another case involved an underweight, developmentally delayed 17-month-old female who presented with fever, loose stools, and failure to gain weight. Physical examination was significant for pallor, hepatosplenomegaly, a rash described as seborrheic in nature, and many boggy, erythematous, and extremely tender swellings on the scalp. Multi-system LCH was confirmed via skull XR and duodenal biopsy. Notably, the patient also had bone marrow and skin involvement. She was first treated with vinblastine and oral prednisolone followed by etoposide, cytarabine, and 2-chlorodeoxyadenosine. The patient was still being considered for stem cell transplant due to bone marrow involvement at the time of publication of the original report [9]. 

In another case report, a five-month-old infant presented with failure to thrive, anemia, and nonspecific elevated inflammatory markers followed by intermittent tachypnea, weight loss, brown cutaneous papules, and greasy, occasionally bloody stools. After initially being treated for food protein-induced allergic proctocolitis without improvement, she was sent to a tertiary hospital and subsequently diagnosed with multi-system LCH. On physical examination, she had a tender, soft tissue mass on her skull and multiple areas of bony tenderness involving her bilateral axilla and lower extremities. Skull XR and skeletal survey revealed diffuse lytic bone lesions involving both the axial and appendicular skeleton. A bone marrow biopsy followed, which confirmed the diagnosis of LCH. Due to the confirmed diagnosis of LCH in the setting of intermittent bloody stools, an esophagogastroduodenoscopy and flexible sigmoidoscopy were performed with biopsies revealing colonic mucosal involvement of LCH. Notably, CNS and pulmonary involvement were later identified on chest CT and brain MRI. The patient was started on weekly vinblastine with prednisone and was continuing treatment at the time of publication of the original report [15]. 

To summarize, our patient did not have GI LCH. Most patients with GI LCH have MSI and multifocal disease and present with more severe and extensive symptoms. 

LCH with vertebral involvement causing compression

When LCH involves the spine, presenting symptoms have been described to range from neck and lower back pain to ankle clonus, extremity weakness, and paraplegia [16]. Of interest, an extraordinarily rare symptom of cord compression associated with LCH is constipation. As far as we know, there has only been one other case report of LCH of the spine (without true GI involvement) that presented with constipation. A 46-year-old female had initial symptoms of back pain, numbness of the lower extremities, urinary retention, and constipation. Initial laboratory workup was unremarkable but MRI was significant for compression fracture of the T6 vertebra with mass effect on the spinal cord. Upon further imaging workup via sella MRI and CT/PET, the patient was found to have increased uptake in the sella as well as in the right parotid gland and renal cortex, which was hypothesized to be additional asymptomatic involvement. The patient was subsequently treated with surgical excision with decompression and screw fixation, followed by systemic chemotherapy [8]. Our patient presented with back pain, which is a common symptom of LCH when it involves the vertebral body.

Limitations

Case reports are generally limited by potential selection and observer biases, which may impact the reliability of the findings. Additionally, the outcome is specific to this patient individually and may not be generalizable. Lastly, no cause-effect relationship can be made. 

## Conclusions

LCH may present as a variety of nonspecific symptoms, such as constipation, making the diagnosis challenging. This case report describes a presentation of LCH of the L3 vertebral body where back pain was masked by constipation and abdominal pain. This case report and review of the literature serves as a reminder to providers of all specialties to maintain a high level of suspicion of rare diagnoses, especially when symptoms have persisted for a prolonged period of time and have not improved with standard treatment. Case reports of uncommon presentations of LCH should continue to be published in order to raise awareness of the rare presentations of this heterogeneous disease.
